# Case Report: Primary extraskeletal osteosarcoma in prostate

**DOI:** 10.3389/fonc.2025.1558053

**Published:** 2025-08-27

**Authors:** Youli Xu, Xing Chen

**Affiliations:** ^1^ Department of Radiology, Cancer Prevention and Treatment Institute of Chengdu, Chengdu Fifth People’s Hospital (The Second Clinical Medical College, Affiliated Fifth People’s Hospital of Chengdu University of Traditional Chinese Medicine), Chengdu, China; ^2^ Department of Orthopedics, Cancer Prevention and Treatment Institute of Chengdu, Chengdu Fifth People’s Hospital (The Second Clinical Medical College, Affiliated Fifth People’s Hospital of Chengdu University of Traditional Chinese Medicine), Chengdu, China

**Keywords:** prostate, extraskeletal osteosarcoma, imaging, CT, MRI

## Abstract

Extraskeletal osteosarcoma (EOS) is an extremely uncommon malignant soft tissue sarcoma that carries a poor prognosis. However, EOS occurring in the prostate is especially rare. The purpose of this case report is to present the radiological features and immunohistochemistry results of the neoplasm in a 72-year-old man. In the case, contrast-enhanced CT and contrast-enhanced MRI clearly showed the primary lesion and the surrounding soft tissue invasion. In our case, massive calcification was seen within the tumor. Despite being regarded as a significant imaging symptom of EOS, calcification only happens in about half of the cases. In our case, positive immunohistochemistry results were included as follows: SATB2 (+), SMA (+), CD99 (+), CK (focal +), Ki-67 (+, hot spots about 40%), P63 (focal +), and Vim (+). Extensive resection is the preferred treatment method for localized lesions. Further adjuvant radiotherapy significantly decreased local recurrences, but chemotherapy did not decrease the risk of systemic recurrence.

## Introduction

Extraskeletal osteosarcoma (EOS) is an extremely rare soft tissue sarcoma with a poor prognosis, characterized by the production of malignant osteoid or bone (or both), excluding osseous origin (1). According to previous case reports and single-center (or two-center) studies, osteosarcoma could occur in a variety of sites in the human body, including the lower limbs, pelvic girdle, upper limb, trunk wall, retroperitoneal, head, uterus, lung, etc., but mainly occurred in the lower limbs (2–4). Still EOS has not been reported in the prostate.

## Case description

A 72-year-old man presented to his doctor a history of progressive dysuria for 2 years and aggravated hematuria for 6 months. The patient had frequent urination, increased nocturia, laborious urination, endless urination, shorter range, gross hematuria, and occasional blood clot symptoms. The laboratory tests of tumor markers in prostate cancer were all shown to be negative.

**Figure 1:**
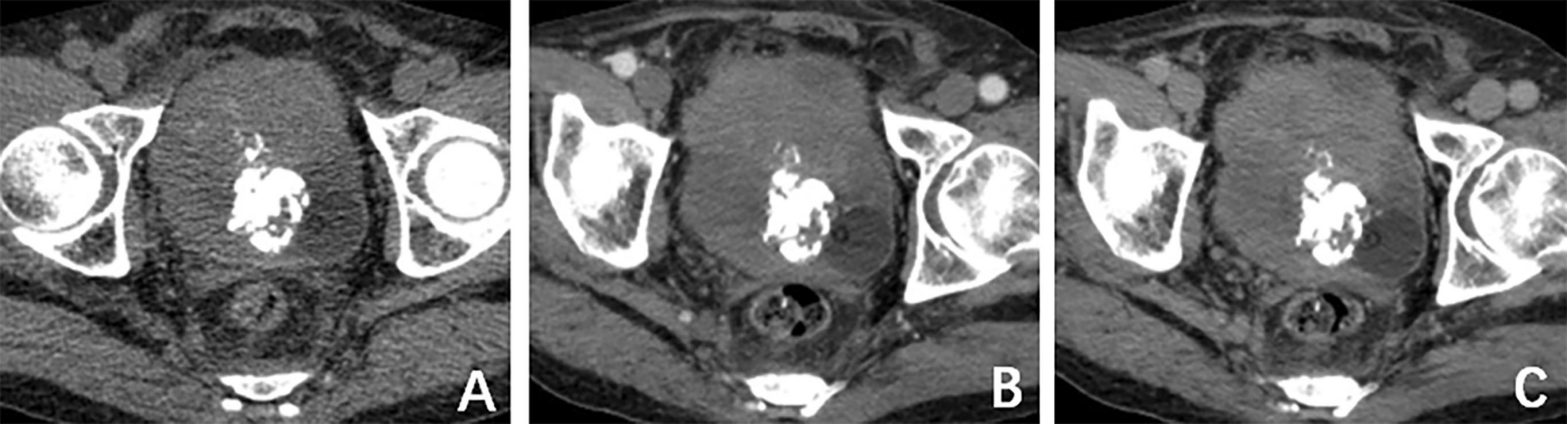
**(A)** Computed tomography (CT) plain scan revealed a large lobulated mass in the prostate area with lumpy calcification. **(B)** CT arterial and **(C)** CT venous phase, respectively. The soft-tissue components of the tumor showed mild fortification in the enhanced CT scan.

**Figure 2:**
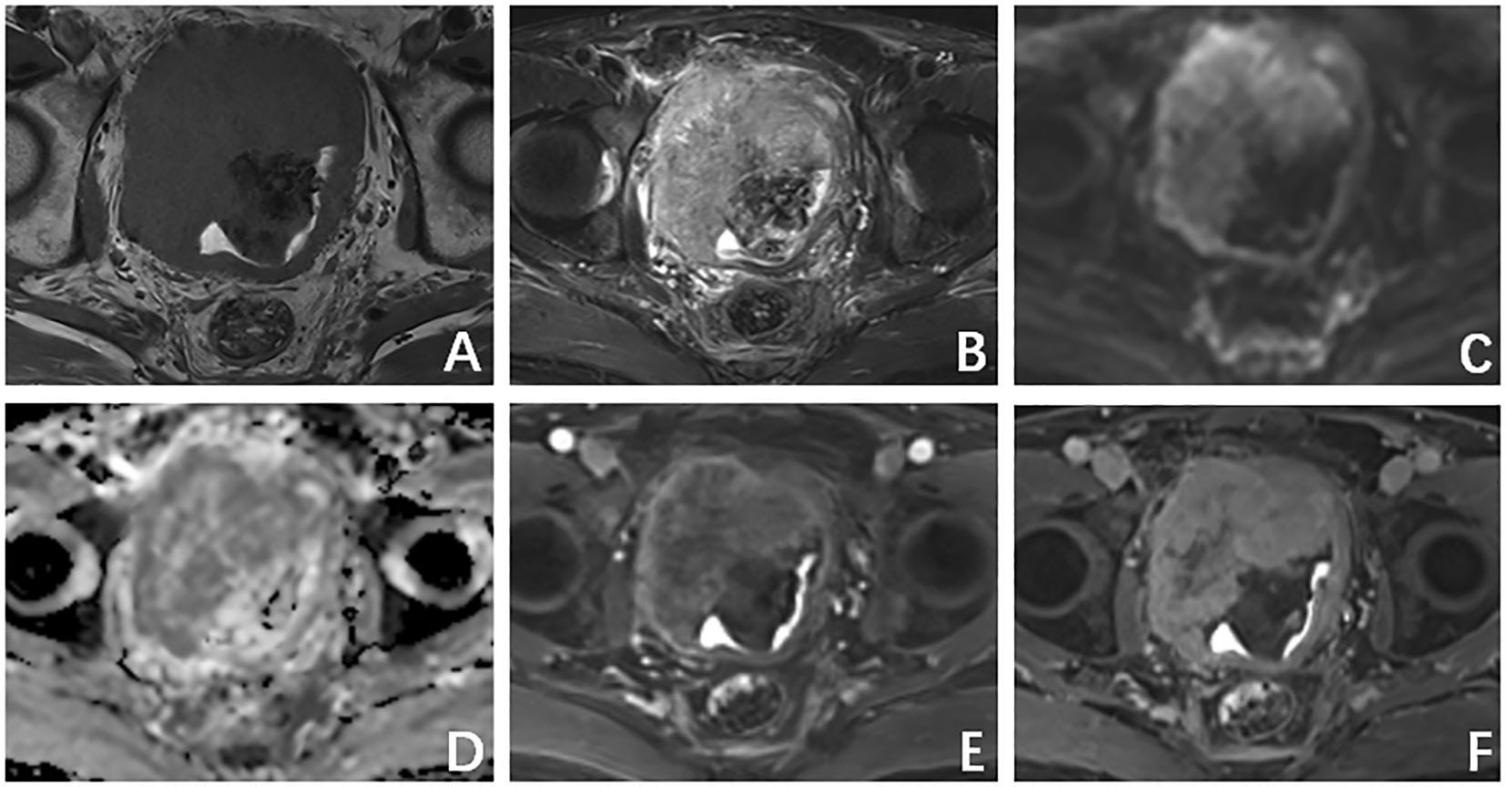
**(A)** T1 weighted image (T1WI) sequence shows an intermediate signal intensity. **(B)** T2 weighted image (T2WI) sequence shows a high and low confounding signal intensity. **(C)** Diffusion weighted imaging sequence shows a high signal intensity. **(D)** Apparent diffusion coefficient (ADC) showed a low-signal-intensity sequence. **(E)** Arterial phase. **(F)** Venous phase shows a progressive inhomogeneous sheet enhancement.

**Figure 3:**
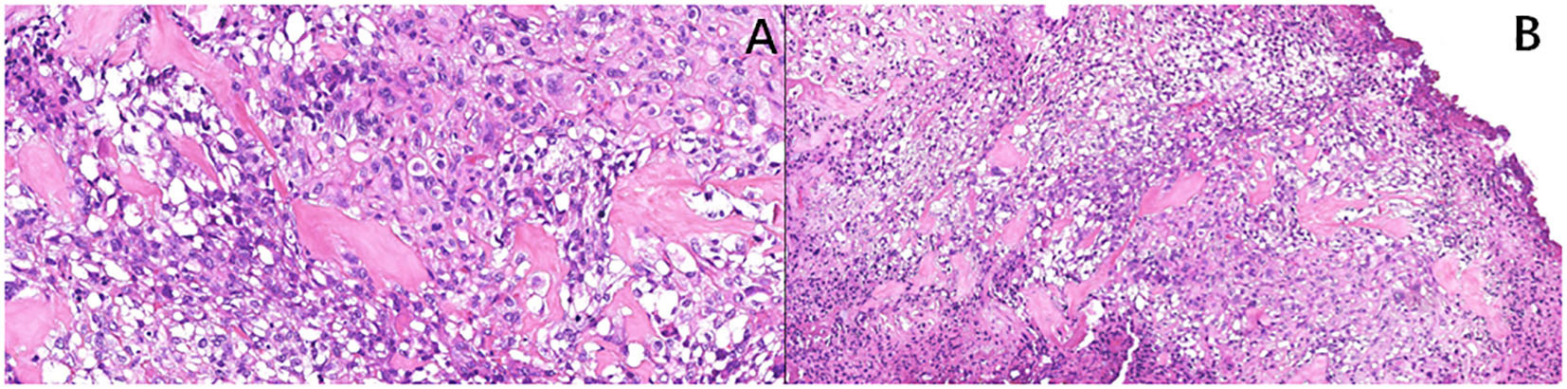
**(A, B)** Histopathology confirmed the diagnosis of extraskeletal osteosarcoma.

A computed tomography (CT) scan of the brain, chest, abdomen, and pelvis revealed a large lobulated mass in the prostate area with lumpy calcification (or ossification) and an ill-defined border. Meanwhile, other site metastases were excluded. The soft tissue components of the tumor showed a mild fortification in the enhanced CT scan (**Figures 1A–C**). The patient underwent 3.0-T magnetic resonance imaging (MRI) examination. The irregular mass of the prostate measuring 80 × 76 × 74 mm showed intermediate signal intensity on the T1 weighted image (T1WI) sequence, high and low confounding signal intensity on the T2 weighted image (T2WI) sequence, high signal intensity on the diffusion weighted imaging (DWI) sequence, low signal intensity on the apparent diffusion coefficient (ADC) sequence, and significantly heterogeneous enhancement on the contrast-enhanced T1WI (CE-T1WI) sequence. The calcification (or ossification) in the mass appeared to have a low signal intensity in all sequences. The mass was poorly defined from the adjacent bladder wall (**Figures 2A–F**). During the operation, the surgeon saw a huge tumor of the prostate protruding into the bladder, raising the posterior lip of the bladder neck, thus resulting in obvious obstruction of the bladder neck. The anesthesia status of the patient was poor during the operation. In addition, the tumor has abundant blood supply, has a large amount of bleeding, and is tough in texture. It cannot be completely resected while ensuring the patient’s good postoperative condition. Therefore, only part of the tumor tissue was resected for biopsy during the operation. After the operation, the patient and the family refused radiotherapy and chemotherapy. The follow-up time of the patients’ survival with the tumor was 13 months. Combining the histomorphology and the immunohistochemical test results, pathologists confirmed the diagnosis as EOS (**Figure 3**). Following are the results of immunohistochemistry: SATB2 (+), SMA (+), CD99 (+), CK (focal +), Ki-67 (+hot spots approximately 40%), P63 (focal +), EMA (-), MyoD1 (-), P504S (-), PSA (-), Vim (+), CD34 (-), Desmin (-), S-100 (-), CK5/6 (-), and CK7 (-).

## Discussion

EOS refers to osteosarcoma that occurs outside bones, which is histologically similar to primary osteosarcoma of bone. However, compared to primary osteosarcoma, EOS occurs in a distinctly older age group (5).

Calcification or ossification is considered to be an important imaging manifestation of EOS, especially thick and lumpy mineralization (4), but calcification or ossification only occurred in approximately 50% of cases in EOS (5). Consistent with Amandine’s study, we similarly observed signal intensity heterogeneity on T1WI, T2WI, and CE-T1WI. In Amandine’s study, 42% of cases presented rim-like peripheral enhancement. However, in our case, the mass showed a progressive inhomogeneous sheet enhancement on CE-T1WI, and the percentage of tumor volume with enhancement was about 75% (2). The most important differential diagnosis for our case is primary prostatic cancer. The serum prostate-specific antigen (PSA), whose threshold of 4 ng/mL has traditionally been used to detect prostate cancer, is the fundamental of prostate cancer investigation and risk stratification but was negative in our study (6). Extensive resection is the preferred treatment method for localized lesions. With the retrospective review at 25 international sarcoma centers of 370 patients, Marilyn’s study results do not support the use of chemotherapy; however, adjuvant radiotherapy demonstrates benefit in patients with locally resectable EOS. Because the osteosarcoma that occurred in the bone is resistant to radiotherapy, Marilyn’s study confirmed that EOS behaves more similar to STS than OS of bone. Considering the resistance of osteosarcoma of bone to radiotherapy, Marilyn Heng’s study confirmed that EOS behaves more similar to soft tissue sarcoma than osteosarcoma of bone (6). In summary, imaging manifestations still lack specificity. Still the clinical and imaging preoperative diagnostic accuracy of external osteosarcoma is poor, and the diagnosis mainly depends on a needle biopsy or an open biopsy.

## Data Availability

The original contributions presented in the study are included in the article/supplementary material. Further inquiries can be directed to the corresponding author.
